# The Differential Diagnosis of Uterine Hæmorrhages.—II

**Published:** 1910-02-12

**Authors:** 


### GYNÆCOLOGY.

### THE DIFFERENTIAL DIAGNOSIS OF UTERINE HÆMORRHAGES.—II.

Having considered the diagnosis of the causes of hæmorrhage in the early months of pregnancy, we now pass on to consider those occurring in the later months. Hæmorrhage at all times in pregnancy is an important symptom, but in the later weeks it becomes doubly important on account of the actual danger to the mother's life which such hæmorrhage may bring about. Apart from new growths of the cervix occurring coincidently with pregnancy, the cause of hæmorrhage at this period is separation of some portion of the placenta from its attachment to the uterus. So far there is no difficulty, but the differential diagnosis of hæmorrhage due to separation of a normally situated placenta from hæmorrhage due to separation of a placenta which is wholly or partially situated in the lower uterine segment is often extremely difficult, and at the same time of great importance to the patient. In other words, we have to distinguish between accidental and unavoidable hæmorrhage. The importance of distinguishing these two classes of hæmorrhage cannot be over-estimated, for, if the blood comes from the separation of a normally situated placenta it is often possible by appropriate treatment to stave off labour, stop the bleeding, and permit the patient to go to full term. In the case of placenta prævia, however, the uterus must be emptied as soon as the condition is diagnosed, for the patient will never be out of danger until the uterus is empty and retracted. No temporising can ever be adopted with safety to the patient in cases of placenta prævia.

Both kinds of bleeding occur more commonly in multiparous patients; both may occur without any history of common accidental injuries, though the patient can usually recall some fall, blow, or emotion to which she attributes the bleeding in accidental hæmorrhage, whilst most patients are equally emphatic that they have had no such accident when the placenta is prævia, and here, too, the bleeding not infrequently commences during sleep. The method by which the blood escapes is of little value in diagnosis, for in each instance the blood may escape equally well during uterine contractions as in the intervals. The chief diagnostic point to be borne in mind is the possibility of feeling the placenta by passing the finger through the internal os. If the placenta is prævia, it can be then reached and felt, its curious stringy character serving to distinguish it from a blood clot. Supposing, however, that the internal os uteri is closed, how then is the diagnosis to be made? This is the real difficulty, for although it is almost always quite easy to force a finger through the internal os, this is just what we do not always want to do in cases of accidental hæmorrhage, for the proceeding is very liable to bring on labour, which may be unnecessary. The only safe rule is to be guided by the prominent signs to be mentioned directly, and, if the hæmorrhage has been at all severe or recurrent, to pass the finger through and make certain. If no placenta can be reached, the case may still be treated as one of accidental hæmorrhage by rest and opium, if the patient's condition suggests this method of treatment. There are, however, certain suggestive signs which may be a valuable aid to diagnosis. In placenta prævia the lower uterine segment may be so filled up by the placenta that the fœtal head is unable to enter it, and so may be found to lie above the brim of the pelvis. It must not be forgotten that in many multiparous women this is the case, if the belly is at all pendulous, apart from any actual obstruction in the lower uterine segment or pelvic brim. It is said that the placenta can sometimes be palpated through the parietes; this must always be a very doubtful point. Per vaginam, however, the lower uterine segment will feel thick and boggy, the presenting part will be obscured, and ballottement will be impossible. Failing to find the placenta in the lower uterine segment by any of these methods, we are justified in considering the bleeding to be accidental and coming from a normal placental site.

The blood, however, does not always escape from the uterus in accidental hæmorrhage, but may remain between the placenta or membranes and the uterine wall. This constitutes concealed accidental hæmorrhage, and its diagnosis is not a matter of very great difficulty. In many cases the bleeding is partly external, whilst the bulk is concealed. There may, however, be no escape of blood externally whatever. In either case the symptoms are important, and the patient may be in a very serious condition. First there are the general symptoms due to loss of blood: pallor, rapid pulse, sighing respiration, coldness of the extremities and clammy perspiration, restlessness. Next we note the symptoms due to over-distension of the uterus by the poured-out blood. This is most important, for it signifies practically paralysis of the uterine muscle. Concealed hæmorrhage is almost impossible to any great extent if good uterine contractions are present. The uterus can be felt to be as hard as a board and tender to the touch, the last being the result of the extreme tension within. The outlines of the fœtus cannot be felt, and the fœtal heart cannot be heard. The cervix is closed as a rule, showing that there have been no contractions: this will not follow in mixed cases of concealed and external hæmorrhage, for in them the cervix may have begun to dilate. These peculiar characters have to be distinguished from tonic contraction of the uterus, in which the same hardness and tenderness of the uterus occur. Here, however, the hardness is due to continuous contraction, and comes on after a long labour, the cervix is always dilated, and there will be no evidence whatever of internal bleeding. Further, the character of the presenting part, and the presence of some obstruction to delivery on the part of the mother or the fœtus will usually point clearly to a definite causation for tonic contraction.

Although not strictly a hæmorrhage of pregnancy post-partum bleeding sometimes calls for differential diagnosis, because it is not always caused by uterine atony or inefficient contraction and retraction. It may be due to trauma—that is to say, to laceration of the cervix, vagina, perinæum, or vulva. The commonest cause is, naturally, atony of the uterus after delivery of the placenta, and its diagnosis is simple enough. The bleeding is sudden, severe, and continuous, while the uterus remains soft and uncontracted. On the other hand, the hæmorrhage from lacerations is continuous, occurring when the uterus is firmly contracted just as it does during relaxation, the only difficulty being to make quite sure that the uterus really is firmly contracted. There is a form of slow, continuous bleeding which occurs with a partially contracted uterus which may give difficulties in diagnosis. It is usually due to retention of some part of the placenta or membranes, and the finger passed into the uterus will recognise a cavity whose walls are partly contracted and not in apposition. The recognition of laceration hæmorrhage depends upon thorough examination of the cervix with a speculum and a good light, drawing down the os with tenacula if necessary. Laceration of the vagina, perinæum, and vulva will be readily detected by inspection.

